# Dental enamel cells express functional SOCE channels

**DOI:** 10.1038/srep15803

**Published:** 2015-10-30

**Authors:** Meerim K. Nurbaeva, Miriam Eckstein, Axel R. Concepcion, Charles E. Smith, Sonal Srikanth, Michael L. Paine, Yousang Gwack, Michael J. Hubbard, Stefan Feske, Rodrigo S. Lacruz

**Affiliations:** 1 Dept. Basic Science and Craniofacial Biology, New York University College of Dentistry, New York, NY 10010, USA; 2 Dept. of Pathology, New York University School of Medicine, New York, NY 10016, USA; 3 Department of Anatomy & Cell Biology, McGill University, Facility for Electron Microscopy Research, Montreal, H3A 2B2, Canada; 4 Faculty of Dentistry, McGill University, Facility for Electron Microscopy Research, Montreal, H3A 2B2, Canada; 5 Dept. of Physiology, University of California, Los Angeles, 53-266 CHS, 10833 Le Conte Avenue, Los Angeles, California, USA; 6 Center for Craniofacial Molecular Biology, Ostrow School of Dentistry, University of Southern California, Los Angeles, California, CSA 135-HSC 9062, USA; 7 Departments of Paediatrics and Pharmacology, The University of Melbourne, Victoria 3010, Australia

## Abstract

Dental enamel formation requires large quantities of Ca^2+^ yet the mechanisms mediating Ca^2+^ dynamics in enamel cells are unclear. Store-operated Ca^2+^ entry (SOCE) channels are important Ca^2+^ influx mechanisms in many cells. SOCE involves release of Ca^2+^ from intracellular pools followed by Ca^2+^ entry. The best-characterized SOCE channels are the Ca^2+^ release-activated Ca^2+^ (CRAC) channels. As patients with mutations in the CRAC channel genes *STIM1* and *ORAI1* show abnormal enamel mineralization, we hypothesized that CRAC channels might be an important Ca^2+^ uptake mechanism in enamel cells. Investigating primary murine enamel cells, we found that key components of CRAC channels (ORAI1, ORAI2, ORAI3, STIM1, STIM2) were expressed and most abundant during the maturation stage of enamel development. Furthermore, inositol 1,4,5-trisphosphate receptor (IP_3_R) but not ryanodine receptor (RyR) expression was high in enamel cells suggesting that IP_3_Rs are the main ER Ca^2+^ release mechanism. Passive depletion of ER Ca^2+^ stores with thapsigargin resulted in a significant raise in [Ca^2+^]_i_ consistent with SOCE. In cells pre-treated with the CRAC channel blocker Synta-66 Ca^2+^ entry was significantly inhibited. These data demonstrate that enamel cells have SOCE mediated by CRAC channels and implicate them as a mechanism for Ca^2+^ uptake in enamel formation.

Ca^2+^ is one of the most abundant elements in mineralized enamel yet the mechanisms allowing the flow of Ca^2+^ from the blood stream to the enamel space during development are poorly understood. Ameloblasts are polarized cells responsible for the regulation of Ca^2+^ transport during enamel formation. These cells form an epithelial barrier restricting the free flow of Ca^2+^ into the enamel layer where hydroxyapatite-like crystals are growing^1^,^2^. Thus ameloblasts handle large quantities of Ca^2+^ and to avoid toxicity, these cells must tightly regulate Ca^2+^ influx and buffering, organellar Ca^2+^ release and sequestration, and Ca^2+^ extrusion. Ameloblasts express Ca^2+^ binding proteins in the cytoplasm and ER^2^,^3^,^4^,^5^,^6^,^22^, with the sarcoplasmic/endoplasmic reticulum Ca^2+^-ATPases (SERCAs) pumps being involved in ER Ca^2+^ sequestration thus contributing to cytosolic Ca^2+^ buffering^7^. Extrusion mechanisms in ameloblasts include plasma membrane Ca^2+^-ATPases (PMCA) as well as K^+^-dependent and K^+^-independent Na^+^/Ca^2+^ exchangers (NCKX and NCX, respectively)^7^,^8^,^9^,^10^,^11^,^12^,^13^,^14^. Despite the critical role of Ca^2+^ in the formation of hydroxyapatite-like crystals, our understanding of the mechanisms employed by ameloblasts to mediate Ca^2+^ uptake and transport remains limited although biochemical data has suggested a “transcytosis” route for Ca^2+^ being channelled across the cell within the ER^2^,^22^,^41^.

Recent evidence gathered by our group first identified one of the components of the Ca^2+^ release-activated Ca^2+^ (CRAC) channel protein STIM1 in murine enamel organ cells from a genome wide study^15^. CRAC channels mediate SOCE, which is an important Ca^2+^ influx pathway in non-excitable and excitable cells that is activated following Ca^2+^ release from the ER^16^,^17^. Depletion of ER Ca^2+^ causes the ER resident proteins STIM1 and STIM2 to interact with ORAI proteins, which form the pore of the CRAC channel in the plasma membrane, enabling localized and sustained Ca^2+^ entry^17^,^18^,^19^. Recent reports have described enamel pathologies in patients with null mutation in *STIM1* and *ORAI1* genes, which are characterized by severely hypo-mineralized enamel^13^,^20^,^21^. These important clinical findings suggest that CRAC channels might be a key mechanism for Ca^2+^ uptake during enamel formation.

Enamel develops largely in two stages, the secretory and maturation stages. The continuously growing rodent incisor is an ideal model to study enamel development as a population of cells from both stages can be identified through life. In the secretory stage, ameloblasts are involved in the synthesis and secretion of enamel-specific proteins, forming an organic template for the growth of thin enamel crystals^1^. During maturation, evidence suggests an increase in the transport capacity of enamel cells, mainly Ca^2+^ and phosphate, which are moved to the extracellular domain to supersaturate the enamel fluid and enable a vast increase in thickness of the enamel crystals^1^,^3^,^15^,^22^,^23^,^24^. The aim of our previous genome wide study was to provide a global overview of the cellular machinery required for the mineralization of enamel^15^. Bioinformatic analysis identified murine *Stim1* and *Stim2* genes as up-regulated transcripts in the maturation stage and we further confirmed these results by Western blot analysis of STIM1 and STIM2 proteins. The present study explores whether secretory stage enamel organ (SSEO) and maturation stage enamel organ (MSEO) cells are equipped with components essential to increases in Ca^2+^ handling capacity, and tests whether Ca^2+^ entry in SSEO and MSEO cells is mediated by CRAC channels.

## Results

### Identification of ER-Ca^2+^ release channels and ER-Ca^2+^ refilling pumps

**Figure 1:**
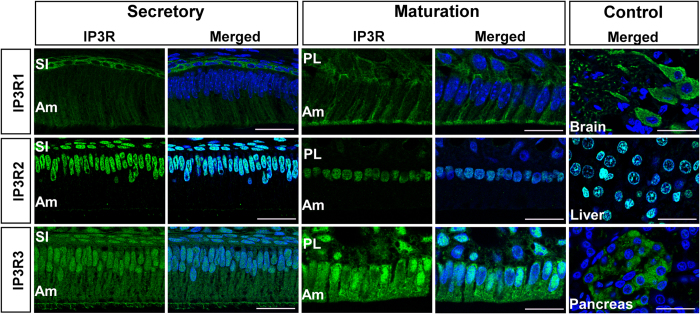
Localization of IP_3_R isoforms in ameloblasts. Upper panel shows IP_3_R1 (green) immunolocalization and nuclear DAPI (blue) staining in rat secretory and maturation ameloblasts. Brain (rat) was used as a positive control. IP_3_R1 is localized intracellularly as also seen in the cells of the *stratum intermedium* adjacent to secretory ameloblasts. In maturation stage, the papillary layer replaces the *stratum intermedium*. Middle panel shows IP_3_R2 (green) immunolocalization and DAPI (blue) in rat secretory and maturation stage ameloblasts. IP_3_R2 is localized to the cell nuclei. Rat liver was used as a positive control. Lower panel shows IP_3_R3 (green) immunolocalization and DAPI (blue) in rat secretory and maturation ameloblasts showing intracellular localization with stronger signal in maturation stage ameloblasts. Pancreas was used as a positive control. Am = ameloblasts; PL = papillary layer; SI = *stratum intermedium* cells. Scale bars in all images = 20 μm.

Transient increases in intracellular Ca^2+^ concentration ([Ca^2+^]_i_) can be mediated by the release of Ca^2+^ from ER stores via IP_3_Rs or/and RyRs^25^. Transcripts of all IP_3_R isoforms were identified in rat secretory stage enamel organ and maturation stage enamel organ cells by RT-PCR, however, RyRs expression levels were negligible compared to those of the IP_3_R transcripts (data not shown). This was also evident by immunofluorescence staining of the RyR proteins which showed very weak signals (Supplementary Fig. S1) and hence we turned our attention to the expression of IP_3_R isoforms as shown in Fig 1. IP_3_R1 reactivity was detected in the intracellular compartment of secretory and maturation stage ameloblasts (Fig. 1, upper panel) but reactivity appeared to increase at the apical pole of maturation stage ameloblasts. IP_3_R2 localized only in the nuclei of secretory and maturation stage ameloblasts (Fig. 1, middle panel). These data are consistent with a previous report on the nuclear localization of IP_3_R2 in endothelial cells^26^. IP_3_R3 showed an intracellular localization in both secretory and maturation stage rat ameloblasts although reactivity was higher in the latter. Weak nuclear staining was also detected for IP_3_R3 in both stages (Fig. 1, lower panel). Other cell types part of the enamel organ which may also participate in transport functions such as *stratum intermedium* (SI) cells in secretory stage, and papillary layer (PL) cells during maturation stage, also showed positive staining for the IP_3_R proteins (see Fig. 1).

**Figure 2:**
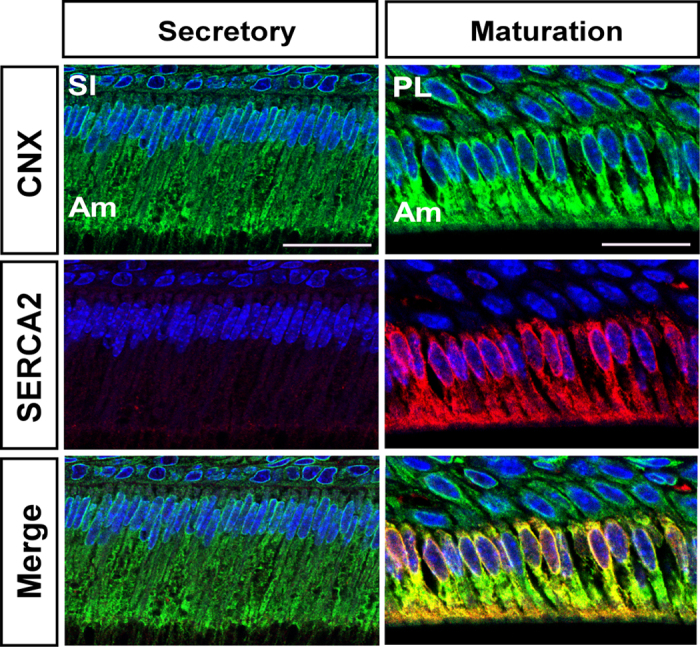
Localization of SERCA2 in ameloblasts. SERCA2 was almost absent in secretory stage ameloblasts but maturation ameloblasts showed strong signals localized to the cytoplasm. SERCA2 expression closely coincides with the localization of the ER as shown by the expression of the ER marker calnexin (CNX). SERCA2 are pumps involved in the transport of Ca^2+^ from the cytosol into the ER lumen. Am = ameloblasts; PL = papillary layer; SI = *stratum intermedium* cells. Scale bars in all images = 20 μm.

Refilling ER Ca^2+^ stores is mediated by SERCA pumps that sequester cytosolic Ca^2+^ into the ER lumen^27^. The murine genes coding for the SERCA isoforms are *Atp2a1*, *Atp2a2*, *Atp2a3*. A previous study had reported on high protein levels for SERCA2 in maturation stage enamel cells^7^. Consistent with those results, here we identified abundant mRNA levels of SERCA2 (*Atp2a2*) by RT-PCR in maturation stage enamel organ cells, which was also the predominant isoform (Supplementary Fig. S2). We therefore investigated SERCA2 localization in ameloblasts by confocal microscopy (Fig. 2). In secretory ameloblasts, SERCA2 signals (red fluorescence) were almost absent, but in maturation stage ameloblasts, SERCA2 was very prominent showing an intracellular localization coinciding with the distribution of the ER as shown by the expression of the ER marker calnexin (CNX). Western blot analysis showed an increase in expression of SERCA2 from secretory to maturation stage (Supplementary Fig. S3). These data confirm that required ER-Ca^2+^ release channels and luminal ER-Ca^2+^ refilling pumps are not only present in both stages of enamel development, but are also up-regulated during the calcium-transport intensive stage (maturation).

### STIM and ORAI expression and localization in ameloblasts

**Figure 3:**
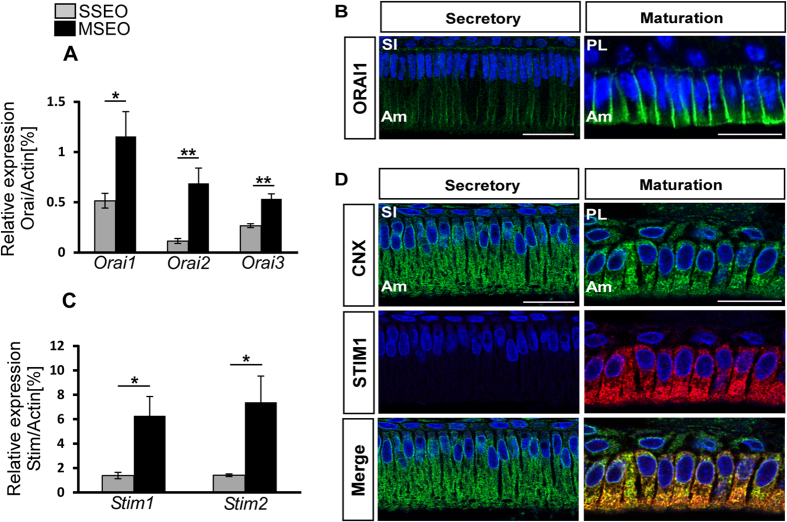
ORAI and STIM1 expression and localization in enamel cells. (**A**,**C**) Relative quantification of *Orai* (1–3) and *Stim* (1–2) transcript levels in SSEO (grey bars) and MSEO (black bars). For RT-PCR, actin was used as a reference gene. Significance was established using two-tailed unpaired Student’s t-test, *(p < 0.05), **(p < 0.01). Each experiment was repeated at least three times. (**B**) Immunofluorescence microscopy showing the cellular localization of ORAI1 (green) in the plasma membrane of mouse secretory and maturation ameloblasts. (**D**) Confocal microscopy showing localization of STIM1 (red) and the endoplasmic reticulum marker calnexin (CNX, green) in rat maturation and secretory ameloblasts. Overlay of STIM1 and CNX is shown in the lower panel (merge). DAPI is shown as blue nuclear staining. Am = ameloblasts; PL = papillary layer; SI=*stratum intermedium* cells. Scale bars in all images = 20 μm.

We then looked for evidence of the molecular components of CRAC channel and identified mRNA expression of all murine *Stim* and *Orai* isoforms in secretory and maturation stage enamel organ cells by RT-PCR (Fig. 3). Comparing mRNA differences between stages we found that all three *Orai* and both *Stim1* and *Stim2* transcripts were more abundant in maturation than secretory stage cells (Fig. 3A,C). These data for *Stim1* and *Stim2* are consistent with our previous gene array and Western blot analysis^15^. As human patients with mutations in *STIM1* and *ORAI1* genes have severely hypo-calcified enamel^13^,^20^,^21^ we focused our attention on the localization of these two proteins in enamel cells. Immunofluorescence studies on paraffin sections of secretory stage ameloblasts showed ORAI1 localized to the basal (supranuclear) and lateral regions of the cells (Fig. 3B). Maturation stage ameloblasts showed stronger ORAI1 immunofluorescence localized at the basolateral pole of the cells consistent with a role of CRAC channels in SOCE (Fig. 3B). In contrast, STIM1 was barely detected in secretory ameloblasts (Fig. 3D) but was strongly expressed in maturation stage cells and showed widespread localization within the cell. STIM1 expression overlapped with the localization of the ER, as shown by the cellular staining of ER marker CNX (Fig. 3D). These data confirm that the molecular components of CRAC channels are expressed in enamel cells. Their cellular localization is consistent with their expected roles as ER Ca^2+^ sensor (STIM1) and pore subunit (ORAI1) of the CRAC channel. We also examined the contribution of STIM2 and investigated its localization by immunoperoxidase staining. STIM2 protein was expressed in both secretory and maturation ameloblasts with stronger signals in maturation (Supplementary Fig. S4).

### Ca^2+^ entry in enamel cells is mediated by CRAC channels

**Figure 4:**
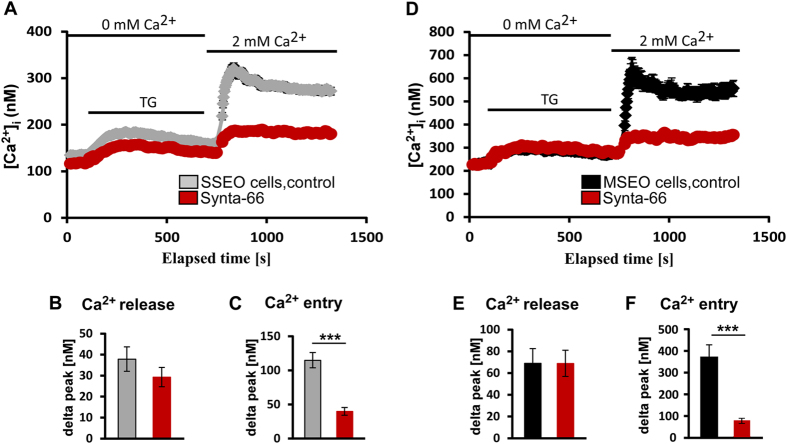
CRAC channels mediate Ca^2+^ entry in enamel cells. (**A**) *Ca*^*2+*^*dynamics in secretory stage enamel organ (SSEO) cells*. SSEO cells were loaded with 5 μM Fura-2 AM and pretreated with Synta-66 (3 μM) (red traces) or left untreated (gray traces). Graphs represent means of [Ca^2+^]_i_ (in nM) calculated from the means of F340/F380 Fura-2 fluorescence ratios recorded in SSEO cells. SSEO cells were stimulated with thapsigargin (1.25 μM) to deplete ER stores resulting in an increase in [Ca^2+^]_i_. Following readdition of extracellular Ca^2+^ (resulting in a final extracellular [Ca^2+^] of 2 mM), we observed a marked increase in [Ca^2+^]_i_ but in Synta-66 treated SSEO cells this [Ca^2+^]_i_ increase was significantly inhibited (p < 0.001, ANOVA). Panels *B* and *C* show delta peak (in nM) of ER- Ca^2+^ release and Ca^2+^ entry in control cells compared to Synta-66 pretreated cells Untreated SSEO cells analyzed: n = 16 (grey bars), Synta-66 pre-treated SSEO cells: n = 8 (red bars). ***(p < 0.001), ANOVA. (**D**) *Ca*^*2+*^ *dynamics in maturation stage enamel organ (MSEO) cells*. Experiments were conducted as described for panel A. Untreated MSEO cells (black traces), Synta-66 pre-treated MSEO cells (red tracings). MSEO cells stimulated with thapsigargin showed an increase in [Ca^2+^]_i_. Following readdition of extracellular Ca^2+^ there was a marked increase in [Ca^2+^]_i_ but not in Synta-66 treated MSEO cells which was significantly inhibited (p < 0.001, ANOVA). Panels *E* and *F* show delta peak (in nM) of ER- Ca^2+^ release and Ca^2+^ entry in control cells compared to Synta-66 pretreated cells. Untreated MSEO cells analyzed: n = 14 (black bars); Synta-66 pre-treated MSEO cells analyzed: n = 8 (red bar). ***(p < 0.001), ANOVA.

Intracellular Ca^2+^ levels were measured by loading secretory and maturation stage enamel cells with the fluorescent Ca^2+^ indicator dye Fura-2-AM. Basal cytosolic Ca^2+^ levels for secretory and maturation stage enamel cells were measured prior to thapsigargin stimulation. We found significantly higher basal cytosolic Ca^2+^ concentrations (p < 0.05, Student’s t-test) in maturation stage enamel cells (~230 nM) compared to secretory stage enamel cells (~135 nM) (Fig. 4, see also Supplementary Fig. S5). Depletion of Ca^2+^ stores activates SOCE mediated by CRAC channels. We thus stimulated enamel cells with thapsigargin (1.25 μM) to block SERCA, a procedure that depletes Ca^2+^ from ER stores through poorly understood ER leaks^28^. Thapsigargin is commonly used for activating SOCE in many cell types^29^,^30^. Thapsigargin was first applied in the absence of extracellular Ca^2+^ resulting in increased levels in [Ca^2+^]_i_ due to Ca^2+^ release from ER stores (Fig. 4A,B). We found that in secretory stage cells, ER Ca^2+^ release increased [Ca^2+^]_i_ by ~40 nM. In maturation stage cells this increase was in the order of ~70 nM.

Ca^2+^ entry was subsequently analyzed upon readdition of extracellular Ca^2+^ to the cells. We identified a significant increase (p < 0.05, Student’s t-test) in [Ca^2+^]_i_ in both cell types demonstrating that enamel cells from secretory and maturation stages have SOCE. This increase in [Ca^2+^]_i_ was however significantly higher in maturation stage enamel cells than in secretory stage cells (p < 0.05, Student’s t-test) (Supplementary Fig. S5). The increase in [Ca^2+^]_i_ following readdition of Ca^2+^ was ~115 nM in secretory stage cells and ~350 nM in maturation stage cells (Fig. 4 and Supplementary Fig. S5). These data are consistent with increased expression levels of STIM and ORAI molecules at the maturation stage (Fig. 3).

CRAC channels are the prototypical SOCE channel. To test whether secretory and maturation stage enamel organ cells have functional CRAC channels, we used a specific pharmacological CRAC channel blocker (Synta-66)^31^ to analyse the effects on SOCE. We found that cells from the maturation and secretory stage pre-treated with Synta-66 showed significantly reduced (p < 0.001) Ca^2+^ influx upon readdition of extracellular Ca^2+^ (Fig. 4). These results indicate that SOCE in enamel cells is mediated by CRAC channels.

## Discussion

Ca^2+^ transport is critical for enamel development and yet the cellular mechanisms associated with Ca^2+^ entry remain a central question in enamel biology. The present study demonstrates that rat secretory stage enamel organ and maturation stage enamel organ cells express SOCE with CRAC channel properties. As Ca^2+^ release from the ER pools is required for SOCE activation, we investigated whether enamel cells are equipped with common receptors involved in ER-Ca^2+^ release and to compare possible differences among them. Inositol 1,4,5-trisphosphate receptors (IP_3_Rs) and ryanodine receptors (RyRs) are the main ER Ca^2+^ release mechanisms^25^,^32^. Immunofluorescence studies showed very weak signals for all RyR isoforms (Supplementary Fig. S1) but not for IP_3_R, revealing differences in cellular localization (Fig. 1). IP_3_R1 and IP_3_R3 showed a cytoplasmic localization whereas IP_3_R2 was found only in the nuclei. These expression patterns are consistent with reports for other tissues^30^,^33^ and although it is not uncommon to identify all IP_3_R isoforms expressed in the same cells, differences in cellular localization have been associated with the performance of various functions. The nuclear localization of IP_3_R2 in secretory and maturation stage ameloblasts suggests that this protein might be involved in nuclear Ca^2+^ signaling as observed in other cells types where this isoform is restricted to the nucleus^34^. The roles of IP_3_R1 and IP_3_R3 are perhaps less clear. IP_3_R1 showed a more limited cytoplasmic localization than the extensive cytoplasmic localization of IP_3_R3 in secretory and maturation stage ameloblasts. The ER has a wide distribution in both secretory and maturation stage ameloblast as observed by calnexin (CNX) signals detected in ameloblasts (Figs 2 and 3) and as also identified by electron microscopy^35^. Thus it appears that both proteins might be in close proximity to, and able to interact with, other Ca^2+^ mobilizing organelles including mitochondria, as these are found in the perinuclear region of ameloblasts^35^ and are up-regulated during maturation^36^. Such mitochondria-ER interactions are well known and play complex roles related to Ca^2+^ buffering, ATP production and other functions^37^. Alternatively, differences between IP_3_R1 and IP_3_R3 might be linked to the generation of more localized Ca^2+^ signals or possibly marking differences in ER compartments in ameloblasts but this needs to be explored further. Regardless, the expression of both of these receptor types produce stronger signals in maturation stage ameloblasts, an expression profile similar to that of the ER Ca^2+^ refilling protein SERCA2 as well as increases in STIM1, STIM2 and ORAI1.

This study identified SERCA2 as the most up-regulated of the three SERCAs in enamel cells showing a wide cytosolic expression (Fig. 2; Supplementary Fig. S2). These data are consistent with a previous report describing this isoform as the predominant SERCA in enamel cells^7^. Although the expression pattern of SERCA2 identified here by immunofluorescence shows low expression in secretory stage ameloblasts, our Western blot analysis of SERCA2 (Supplementary Fig. S3) as well as Northern blot and Western blot analysis previously reported^7^ reveals that SERCA2 is indeed expressed at lower levels during the secretory stage. Additionally, our Fura-2 data in secretory stage enamel cells showed increased [Ca^2+^]_i_ levels after blocking SERCA with thapsigargin indicating that despite its low abundance, the function of SERCA is not absent at this stage.

Abundant evidence suggests that the requirement to transport Ca^2+^ increases during the maturation stage of amelogenesis^1^,^2^. In keeping with these studies we found that basal cytosolic Ca^2+^ levels were significantly higher in maturation stage enamel cells than in secretory stage cells. Our quantification of basal [Ca^2+^]_i_ levels in enamel organ cells using Fura-2 showed ~135 nM of [Ca^2+^]_i_ in secretory stage cells whereas in maturation stage cells levels were ~230 nM. To our knowledge this is the first study that quantitates Ca^2+^ levels in enamel cells. Differences in basal cytosolic Ca^2+^ between these cell types may be related to differences in cytosolic Ca^2+^ buffering, extrusion mechanisms, or a combination of these. This elevation during maturation may arise as the requirements for Ca^2+^ availability increase so that it can be more easily extruded.

Upon readdition of extracellular Ca^2+^ both cell types showed a significant increase in [Ca^2+^]_i_ albeit this increase was markedly higher in maturation stage cells. Despite this marked increase during maturation and the known crystal growth expansion that also occurs at this stage, nucleation events leading to the formation of thin crystals in the secretory phase are still likely to require an active Ca^2+^ transport system. This growth requirement is in keeping with the increase in [Ca^2+^]_i_ observed in secretory stage cells (Fig. 4). Despite these differences in Ca^2+^ dynamics between cell types, evidence that both showed Ca^2+^ release after thapsigargin stimulation followed by Ca^2+^ entry upon readdition of extracellular Ca^2+^ indicates that both have SOCE. Importantly, Ca^2+^ entry evoked in enamel cells from both stages treated with the specific CRAC channel blocker Synta-66 was significantly inhibited compared to control cells thus identifying CRAC channels as the SOCE type in enamel cells (Fig. 4). Given that ameloblasts form an epithelial barrier limiting the free flow of Ca^2+^, our data on the cellular localization of STIM1 and ORAI1 (Fig. 3B,D), together with our Fura-2 analysis using a pharmacological CRAC channel inhibitor, strongly suggest that CRAC channels are important mediators of Ca^2+^ entry into enamel cells. The identification of severely hypo-calcified enamel in patients with mutations in *STIM1* and *ORAI1* genes^13^,^20^,^21^ supports the functional role of CRAC channels in enamel development.

The CRAC channel inhibitor used here (Synta-66) significantly reduced Ca^2+^ influx into enamel cells although a small increase in [Ca^2+^]_i_ was still detected (Fig. 4). This small Ca^2+^ increase might be linked to lack of efficient inhibition of this compound in the enamel cells, or it may alternatively indicate that other Ca^2+^ entry channels such as TRPC (putatively linked with SOCE channels^38^,^39^) are active in enamel cells. However our previous genome wide analysis surveying rat enamel organs did not identify the expression of TRPC channels in enamel cells using this methodological approach^15^, and no reports in the literature have thus far linked TRPC channels with abnormal enamel phenotypes.

Data shown here combined with previous studies identifying luminal ER Ca^2+^ buffering proteins^2^,^3^,^22^ indeed highlight the potential role for the ER in enamel cells as an essential component in Ca^2+^ transport enabling us to refine hypotheses concerning Ca^2+^ transport in enamel formation. In enamel biology, the calcium transcytosis hypothesis of Hubbard^2^,^22^,^41^ has gained recognition for several years. In this model, it is recognized that there is limited evidence for a paracellular route for Ca^2+^ movement in enamel epithelium. Instead, an active transport system is more likely^5^,^6^,^7^ through a transcellular route which requires three mechanistic steps: Ca^2+^ entry, transit and extrusion (reviewed in ref. 6). A classical Ca^2+^ transcellular model is the “calbindin-ferry” dogma where cytosolic Ca^2+-^binding proteins (calbindins) act as shuttles of Ca^2+^ across cells. However, the demonstration that calbindin28 kDa-null mice lack a dental phenotype^40^, together with biochemical data indicating that all three calbindins (calbindin 28 kDa, calbindin 9 kDa and calbindin 30 kDa) are not up-regulated during the maturation stage suggests that they are not essential in enamel formation^41^. In contrast, the extensive distribution of the ER in enamel cells, together with the localization of STIM1 and ORAI1 in rat maturation ameloblasts described here which enables multiple Ca^2+^ entry points, supports the role of ER as a key component for Ca^2+^ transport as previously proposed^2^,^22^. Support for this calcium transcytosis model comes from the studies on polarized pancreatic acinar cells where it has been shown that the ER functions as a lumenally continous compartment allowing the movement of Ca^2+^ from the base to the apex^42^,^43^. This might also be the case in ameloblasts but this hypothesis requires further testing.

**Figure 5:**
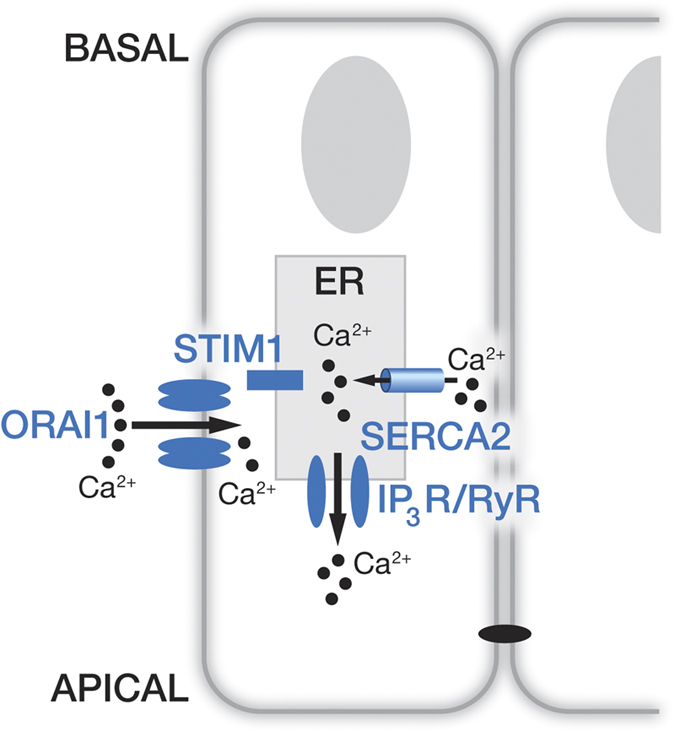
Schematic model representing calcium entry in enamel cells. Working model for Ca^2+^ uptake by enamel cells showing maturation stage ameloblasts forming a cell barrier joined by tight junctions at the apical pole. In the endoplasmic reticulum (ER) we find that enamel cells express the sarco/endoplasmic reticulum SERCA2 as the main Ca^2+^ refilling pump. Inositol 1,4,5-trisphosphate receptors (IP_3_R) and ryanodine receptors (RyR) are also identified as release channels with the former likely being the active release system. STIM1 has a wide distribution throughout the ER and ORAI1 is found in the plasma membrane of enamel cells. As Ca^2+^ pools are depleted in the ER, STIM1 clusters enable Ca^2+^ entry via the ORAI1 channel.

The data presented here allow us to propose a CRAC channel-regulated Ca^2+^ entry model in enamel cells as shown in Fig 5. Concerning the extrusion step, our recent data identifying the exchanger NCKX4 in enamel^12^ and reports on severe enamel pathologies found in patients and mouse models with NCKX4 deficiencies^14^, strongly suggests a key role for this exchanger in Ca^2+^ extrusion during enamel mineralization.

Our work provides the first functional evidence for Ca^2+^ uptake via CRAC channels in primary enamel cells. These data together with the localization of STIM1 and ORAI1 throughout the ameloblasts highlights the possibility that the ER may act as a key organelle enabling Ca^2+^ transport across the cells as held by the calcium transcytosis model. These data greatly advance our understanding of the mechanism regulating Ca^2+^ transport during enamel formation, and warrant further investigations into the role of SOCE in enamel cell signaling and calcium transport.

## Methods

### Tissue Dissections and Real-Time PCR (RT-PCR)

All animal protocols were approved by the Animal Care and Use Committee, and are in full compliance with Federal, State, and local laws and institutional regulations. Animals were maintained following NIH guidelines. Sprague Dawley rats or C57BL/6 mice were used as detailed below for each experiment. The use of rats was preferred over mice for the isolation and processing of enamel organ cells for Fura-2 AM analysis, RT-PCR and Western blot as previous studies had reported on anatomical landmarks in rats that allow us to independently isolate secretory from maturation stage enamel organs and because the larger size of rats makes this procedure easier^44^,^45^. In one instance, we used paraffin embedded mouse tissues for immunohistological analysis as the antibody had been widely used in mouse tissues but had not been tested in rats. This was the case for the ORAI1 antibody (see below). Rats and mice do not differ in the histological development of the enamel in any significant way. Rats (100–160 gram) were euthanized and their mandibles were immediately dissected out, then the surrounding soft tissues were removed. The bulk of the dissected enamel organ during the secretory stage is composed by ameloblasts but other adjacent cell types such as *stratum intermedium* cells may also be present. During maturation, papillary layer cells replace the *stratum intermedium*. Total RNA was extracted from rat secretory and maturation stage enamel organ cells and control tissues using RNeasy^R^ Micro Kit (Qiagen, USA) according to the manufacturer’s specifications. Reverse-transcribed PCR was performed using iScript^tm^ cDNA Synthesis Kit (Bio-Rad, USA). RT-PCR amplifications were set up in a total volume of 20 μl using 0.7 μg of cDNA, 250 nM forward and reverse primer using SsoAdvanced^TM^ Universal SYBR Green Supermix (BioRad, USA). Cycling conditions were as follows: initial denaturation at 95 °C for 3 min, followed by 39 cycles of 95 °C for 10 sec, 58 °C for 30 sec. Specificity of PCR products was confirmed by analysis of a melting curve. RT-PCR amplification were performed on a CFX Connect^TM^ Real-Time System (Bio-Rad, USA) and all experiments were done in triplicate. The housekeeping gene beta-actin was amplified to standardize the amount of sample RNA. Relative quantification of gene expression was achieved using the ΔCT method. Primer sequences used can be found in the Supplemental Data section.

### Immunostaining and Western blot

For immunohistochemical analysis, rats and mice at 10 days postnatal were euthanized. Isolated mandibles stripped of soft tissues were immersed in formalin overnight at 4 °C and decalcified in 4.13% EDTA (pH 7.3) for 7–10 days and washed. Appropriate positive control tissues were also collected and processed as for dental tissues but without the decalcification step. Samples were embedded in paraffin for sectioning (5 μm thick). After deparaffinization, antigen retrieval was performed by cooking the slides for 20 min in 10 mM citric buffer (pH 6.0) followed by blocking for 1 hr with Dako Diluent (Dako, USA). The following antibodies and dilutions were used: anti-STIM1 (1:200, Sigma-Aldrich), anti-calnexin (a marker for ER) (1:1000, Abcam), anti-ORAI1 (1:50, described in^20^) as well as anti-IP_3_R1 (1:50, Novusbio), anti-IP3R2 (1:100, Sigma-Aldrich), anti-IP3R3 (1:1000, Abcam), anti-SERCA2 (1:200, Abcam), anti-RyR1 (1: 500, LSBio), anti-RyR2 (1:200, Sigma-Aldrich) and anti-RyR3 (1:300, Abcam). Sections were incubated overnight at 4 °C. Negative controls were provided by incubating sections with Dako Diluent lacking primary antibody. After washing with PBS, sections were incubated for 30 min at room temperature using appropriate secondary antibodies from Invitrogen at 1:800 (Anti Rabbit IgG AlexaFluor488 and/or Anti-Mouse IgG Alexa Fluor555). After washing, slides were mounted with a coverslip using Prolong Gold Mounting Media (Invitrogen, USA) containing DAPI. Images were obtained using a Leica TCS SP5 II confocal microscope or a Nikon Eclipse 2000TE. The immunolocalization of Stim2 was performed by incubating with an anti-Stim2 antibody (1:50, Sigma-Aldrich) using Vectastain ABC Kit (Vector Laboratories) and counterstaining with haematoxylin before mounting the slides with Vecta Mount Mounting Media (Vector Laboratories). Samples were imaged using a Nikon Eclipse E600 microscope with NIS Elements BR3.2 (Nikon) software. Western blot analysis of SERCA2 was performed as described^45^ using the following antibodies: SERCA2 (1:1000, Abcam), and actin (1:2000, Santa Cruz).

### Cell Isolation

Rats (100–160 gram) were euthanized, mandibles were dissected out and the surrounding soft tissues removed. Isolated mandibles were transferred to Hanks solution (GIBCO) containing 1% Antibiotic-Antimycotic and kept on ice at all times. Isolated SSEO and MSEO cells were treated with 0.15% collagenase (Roche, Tokyo, Japan) in PBS for 1 hr at 37 °C in a 5%-CO_2_ incubator followed by Trypsin/EDTA (Gibco, Life Technologies, USA) for 10 min at 37 °C in a 5%-CO_2_ incubator and manual disruption. Reaction was stopped by adding DMEM (Gibco by Life Technologies, USA) containing 30% fetal bovine serum (FBS), 1% penicillin/streptomycin, 1% glutamine. For intracellular Ca^2+^ concentration ([Ca^2+^]_i_) measurement, isolated SSEO and MSEO cells were cultured in X-Vivo^TM^ 15 (Lonza, USA) cell media containing 10% FBS, 1% penicillin/streptomycin, 1% glutamine and maintained at 37 °C in a 5%-CO_2_ incubator. Cells were used within 24 hrs after isolation.

### Intracellular Ca^2+^ measurements

Secretory stage enamel organ (SSEO) cells and maturation stage enamel organ (MSEO) cells were kept in media for 4 hrs at 37 °C in a 5%-CO_2_ incubator. To determine intracellular Ca^2+^ concentrations ([Ca^2+^]_i_), cells were loaded with Fura-2-AM (5 μM, Life Technology Invitrogen, USA) for 30 min at room temperature. Cells were then washed with X-Vivo^TM^ 15 cell media containing 10% FBS, 1% penicillin/streptomycin and 1% glutamine. Finally, cells were dispensed into 96-well black clear bottom plates. Fura-2 emission was detected at 510 nm after excitation at 340 nm and 380 nm using a FlexStation3 plate reader (Molecular Devices, USA)^46^. The ratio (R) of Fura-2 emission following excitation at 340 nm (F340) and 380 nm (F380) were calculated for each time point. [Ca^2+^]_i_ was estimated from calibration curves by using the Calcium Calibration Buffer Kit#1 (Molecular Probes, Life technologies, USA) using the following equation: [Ca^2+^]_i_ = K_d_ x ((R-R_min_)/(R_max_-R)) x (F380_max_/ F380_min_) as described by Grynkyewicz^46^. K_d_ is the dissociation constant of Fura-2 (K_d_ = 156 nM); R is the ratio of Fura-2 emission following excitation at 340 nm divided by the Fura-2 emission following excitation at 380 nm; R_min_ was measured in nominally Ca^2+^ free buffer; R_max_ was measured at saturating Ca^2+^ concentration (39 μM); F380_max_ is the fluorescence intensity after excitation at 380 nm in Ca^2+^ free buffer; F380_min_ is the fluorescence intensity at saturating Ca^2+^ concentration (39 μM). All experiments were performed at 25 °C. In order to measure SOCE, intracellular Ca^2+^ stores were depleted by inhibiting SERCA activity with thapsigargin (1.25 μM, Sigma-Aldrich, USA) in cells kept in Ca^2+^ free Ringer solution followed by readdition of an equal volume of Ca^2+^-containing Ringer solution at the times indicated to a final concentration of 2 mM Ca^2+^. For some experiments, SSEO and MSEO stage cells were treated with the CRAC channel inhibitor Synta compound 66 (Synta-66, 3 μM)^31^ for 30 min before initiating the experiment and then Synta-66 was added to all the solutions used during the experiment. Buffer solutions contained (in mMol/l): 125 NaCl, 5 KCl, 1.2 MgSO_4_, 32.2 Hepes, 2 Na_2_HPO_4_, 4 CaCl_2_, and 5 glucose at pH 7.4 (extracellular Ca^2+^ buffer) or 125 NaCl, 5 KCl, 1.2 MgSO_4_, 2 Na_2_HPO_4_, 32.2 Hepes, 0.5 EGTA, 5 glucose, pH 7.4 (Ca^2+^-free buffer). Ca^2+^ data were acquired using SoftMax Pro software (Molecular Device, USA) and analyzed using GraphPad Prism v.5. Cells were analyzed for baseline [Ca^2+^]_i_, increase in [Ca^2+^]_i_ after ER depletion and SOCE. The area under the curve (A.U.C.) following Ca^2+^ influx was analyzed using GraphPad Prism v.5.

### Statistics

Data are provided as mean ± SEM, *n* represents the number of independent experiments. Differences were tested for significance using Student’s unpaired two-tailed *t*-test or ANOVA. *P*<0.05 was considered statistically significant.

## Additional Information

**How to cite this article**: Nurbaeva, M. K. *et al.* Dental enamel cells express functional SOCE channels. *Sci. Rep.* **5**, 15803; doi: 10.1038/srep15803 (2015).

## Supplementary Material

Supplementary Information
