# Supplementary material for: Dental enamel cells express functional SOCE channels
Source: Sci Rep. 2015 Oct 30;5:15803. doi: 10.1038/srep15803 (PMC4626795; doi:10.1038/srep15803)
Supplement: Supplementary Information [file srep15803-s1.pdf]

Dental enamel cells express functional SOCE channels

Meerim K. Nurbaeva, Miriam Eckstein, Axel R. Concepcion, Charles E. Smith, Sonal Srikanth, Michael L. Paine, Yousang Gwack , Mike J. Hubbard, Stefan Feske & Rodrigo S. Lacruz

Supplementary Information:

Supplementary Table S1: Primer sequences used for RT-PCR

For amplification the following primers were used (5`->3`orientation):

Gene	Fw primer	Rev primer
Itpr1	ATCACCATCATGCAGCCTAT	TCTCCAGGGTTTGGTTGATA
Itpr2	GTCCGATATCCAGTGTTTGC	TCATGCAACTGCTGGTAGAA
Itpr3	TGAAGTGGGCCATAACATCT	GGGACGTGTGGTTCTCATAG
RyR1	AGCGCTCTATGTTTGACCAC	TGACACAACCTTTTCCAGGT
RyR2	CCTTTGAGAGGCAAAACAAA	TGCACTCGAAGGAGGTAGTC
Orai1	GGTGAAGTTCTTACCGCTCA	ACGGCAAAGACGATAAACAC
Orai2	AATGGGACATACTGCCAAGC	TGCCAAACAAACAAACCAAA
Orai3	ACCTCACACCATCATCTGCT	AGTTCCTCCAGTTCCTGCTT
Stim1	CTGTCTCTGCTGTCCCAGTT	TCCATAGAACAATCCCCAGA
Stim2	ATGCACCAGCTCTCTAGTGG	TTGATGGCTTTTTGCTTTTC
Atp2a1	CAGCCTGTCTGAGAACCAGT	CTCCAGATAGTTCCGAGCAA
Atp2a2	GAAGTCTGCCTTCTGTGGAA	ACTTCTCCAATGGGTGCA
Atp2a3	GATCACAGGGGACAACAAAG	ATGGCGGTGATCTCATTAAA
Actin	CACACTGTGCCCATCTATGA	CCGATAGTGATGACCTGACC

Supplementary Figure S1. Weak immunofluorescence signals of RyRs in secretory and maturation stage ameloblasts.

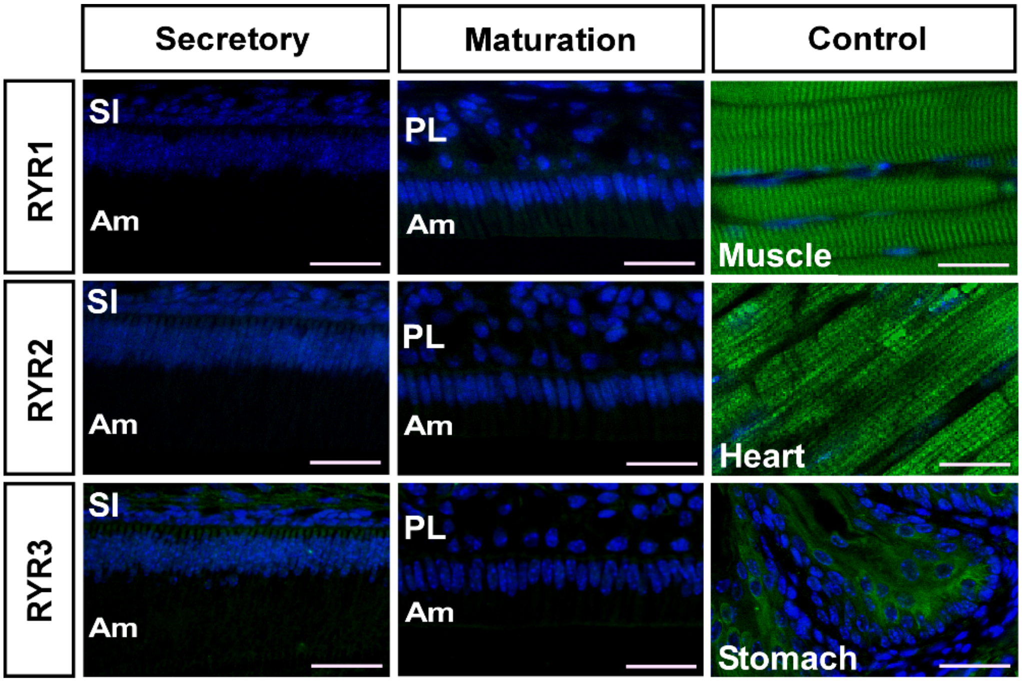

Figure S1. RyRs isoforms in ameloblasts. RyR isoforms showed very weak immunofluorescence signal in secretory and maturation ameloblasts. Upper panel shows RyR1 (green) immunolocalization and nuclear DAPI (blue) staining in secretory and maturation ameloblasts. Skeletal muscle was used as a positive control. Middle panel shows RyR2 (green) immunolocalization and DAPI (blue) in secretory and maturation stage ameloblasts. Heart muscle was used as a positive control. Lower panel shows RyR3 (green) immunolocalization and DAPI (blue) in secretory and maturation ameloblasts. Stomach was used as a positive control. Am= ameloblasts; PL= papillary layer; SI=*stratum intermedium* cells. Scale bars: RyRs: secretory, maturation= 20 µm.

Supplementary Figure S2. SERCA isoform expression in enamel cells.

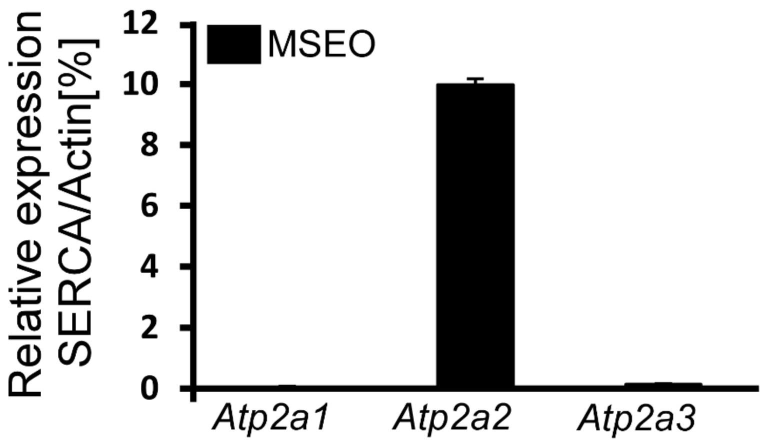

Fig S2. RT-PCR showing mRNA expression levels of SERCA isoforms (coded by the genes *Atp2a1-3*) in rat maturation stage enamel organ cells. SERCA2 is the dominant isoform. Actin was used as a reference gene. Significance was established using two-tailed unpaired Student's t-test, ($p<0.001$). Experiment was repeated three times.

Supplementary Figure S3. Western blot analysis of SERCA2 in enamel organ cells.

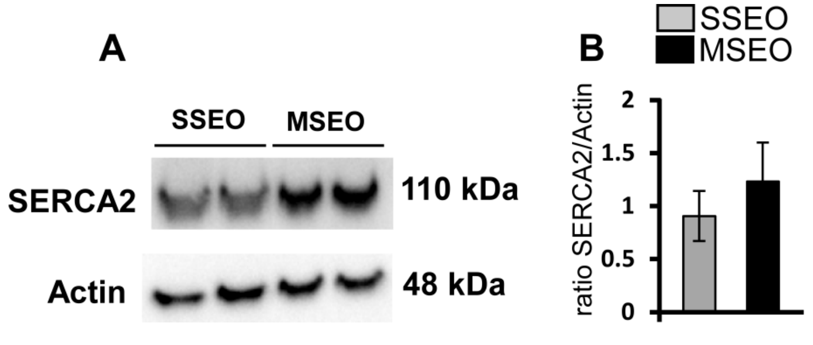

Figure S3. **SERCA expression pattern in enamel organ cells**. **A)** SERCA2 protein expression pattern was assessed in secretory and maturation stage enamel organ cells (SSEO and MSEO). Actin was used as loading control. Both secretory and maturation stage enamel organs show SERCA2 expression but with a noticeable increase in maturation. These data are consistent with previous reports[7]. **B)** Quantification of SERCA protein expression normalized to Actin in rat SSEO and MSEO cells using Image J software to measure the optical density of bands in Western blot experiments. Error bars represent ± SEM.

Supplementary Figure S4. Immunolocalization of STIM2 in secretory and maturation stage ameloblasts.

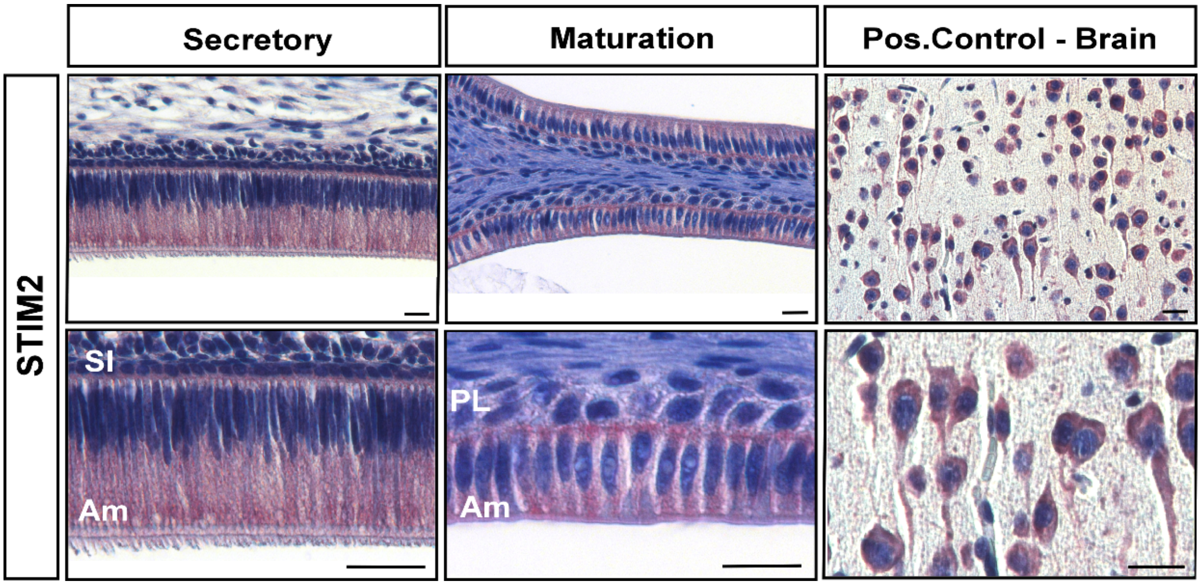

Figure S4. Localization of STIM2 in ameloblasts. Paraffin sections of 10 day old rat mandibles fixed in 4% paraformaldehyde were used to assess the localization of STIM2 by immunoperoxidase staining using anti-STIM2 antibody (Sigma Cat#HPA036933). Top panel is overview (20x) of secretory and maturation stage ameloblasts. Bottom panel shows close up of the same areas (40x). Both cell types show intracellular localization of STIM2. Brain was used as control tissue. Scale bars: 20μm in all images.

Supplementary Figure S5. Functional SOCE in rat SSEO and MSEO cells

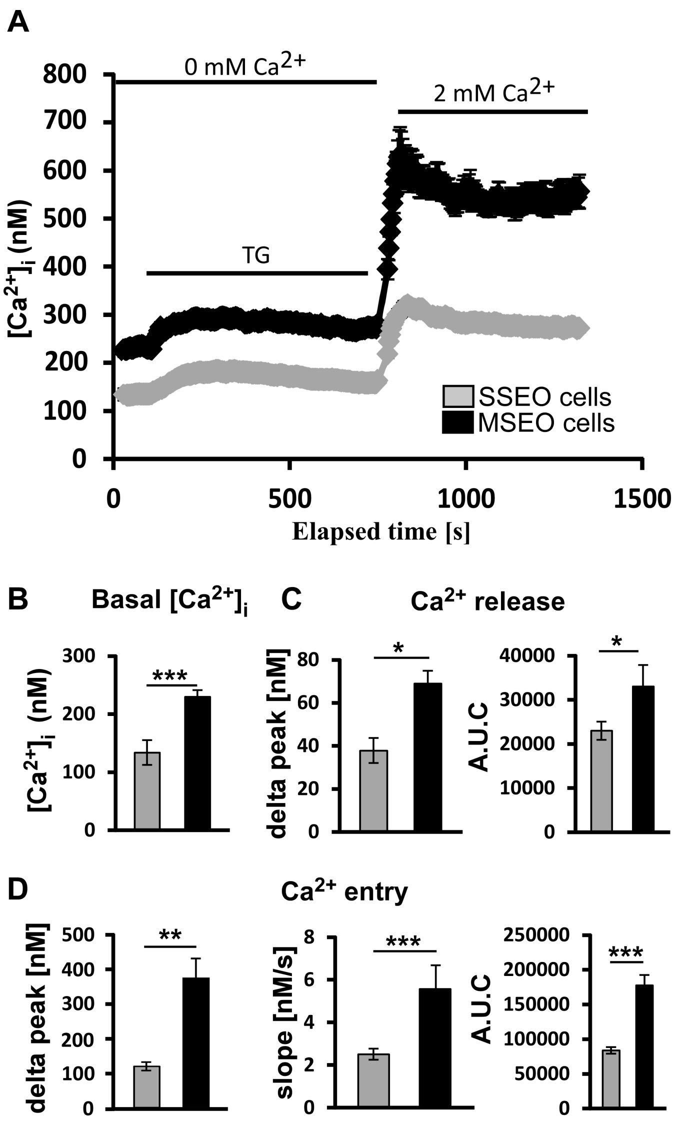

Figure S5. A) Representative original tracings showing Fura-2 fluorescence ratios (340/380 nm) in Fura-2/AM loaded SSEO (grey tracings) and MSEO (black tracings) cells. Experiments were carried out first in zero extracellular Ca^{2+} for about 100 secs before addition of thapsigargin (TG) (1.25 µM) where indicated to block SERCA and enable the release of Ca^{2+} from the stores leading to a rise in $[Ca^{2+}]_i$. As $[Ca^{2+}]_i$ declined, extracellular Ca^{2+} (to a final concentration of 2 mM) was added to assess Ca^{2+} entry through SOCs. B, C, D) Mean (± SEM) of the Fura-2 ratio basal $[Ca^{2+}]_i$ (B) and peak (left C, D), slope (right D) and A.U.C (right C,D) of the change in Fura-2 fluorescence following addition of TG (1.25 µM) in the absence (Ca^{2+}-release, C) and in the presence (Ca^{2+} entry, D) of extracellular Ca^{2+} in rat SSEO (n= 16, grey bars) and MSEO (n= 14, black bars). * ($p<0.05$), ** ($p<0.01$), *** ($p<0.001$), two-tailed unpaired Student's t-test. We have also quantified the area under the curve following thapsigargin stimulation and SOCE. No differences were found between this method and the delta peak. It is also noticeable that the slope of Ca^{2+} entry (speed of Ca^{2+}entry) uptake in MSEO cells was significantly greater ($p<0.001$) than in SSEO cells.

Supplementary Figure S6. Quantification of changes in $[Ca^{2+}]_i$ in SSEO and MSEO control cells relative to Synta-66 SSEO and MSEO treated cells.

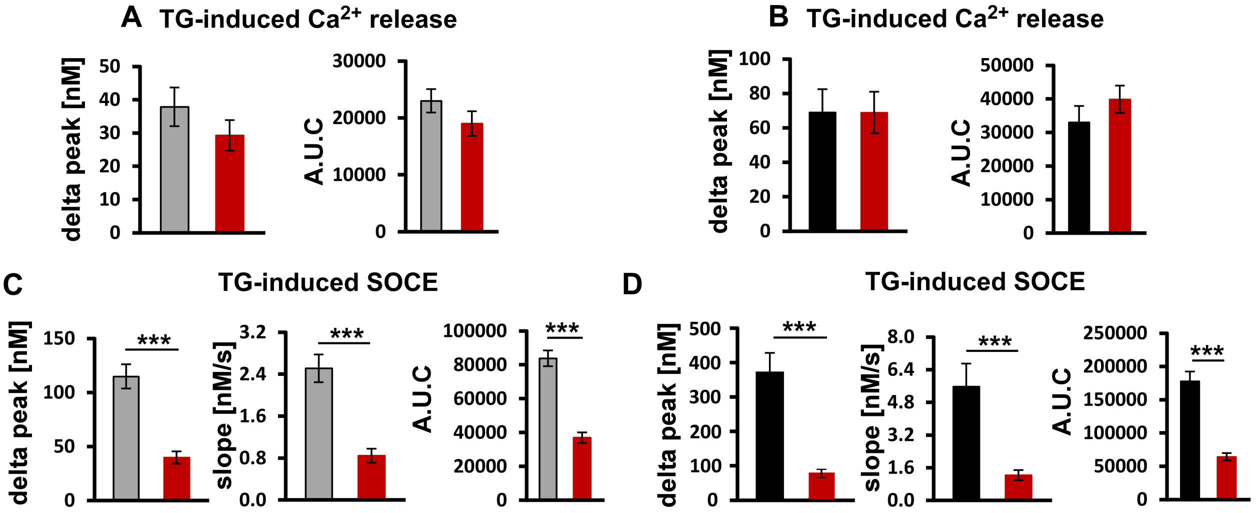

Figure S6. In addition to showing statistics for delta peaks after thapsigargin stimulation and SOCE, we also calculated area under the curve (A.U.C.) for secretory and maturation stage enamel cells. The delta peak following thapsigargin stimulation showed similar changes to those identified by quantifying the A.U.C. The same was observed for SOCE after re-addition of extracellular Ca^{2+}. Untreated SSEO cells analyzed: n=16 (grey bars), Synta-66 pre-treated SSEO cells: n=8 (red bars). *** (p<0.001), ANOVA. Untreated MSEO cells analyzed: n=14 (black bars); Synta-66 pre-treated MSEO cells analyzed: n=8 (red bar). *** (p<0.001), ANOVA.
